# Supplementary material for: Patterns of Intron Gain and Loss in Fungi
Source: PLoS Biol. 2004 Nov 30;2(12):e422. doi: 10.1371/journal.pbio.0020422 (PMC532390; doi:10.1371/journal.pbio.0020422)
Supplement: Table S1 — Also available at http://genes.mit.edu/NielsenEtAl/. (4.3 MB ZIP). [file pbio.0020422.st001.zip › NielsenEtAl/html/1447.html]

AN2862.1.NCU00243.1.MG05427.1.FG06627.1


```
 CLUSTAL W (1.82) Multiple Sequence Alignments - Introns Inserted


Sequence 1: MG05427.1	238 aa
Sequence 2: FG06627.1	241 aa
Sequence 3: NCU00243.1	248 aa
Sequence 4: AN2862.1	246 aa
Alignment Length: 253 aa
Number Identitical Residues: 130 aa
Alignment Score (without introns) 6258


MG05427.1 	MGESR2---------LLQLNITKVEQCGTG2AALCQVFDSIFL~DVPMSRVKFNVNSEYA
NCU00243.1	MGESR2QELLQWINSLLQLNLTKVEQCGTG2AALCQVYDSIFG~DVPMSRVKFNVNSEYA
FG06627.1 	MGESR2QELVAWLNSLLQLNITKVEQCGTG2AALCQVFDSIYM~DVPMSKVKFNVNSEYL
AN2862.1  	MGESR2QELLAWLNNLLQLNMTKIEQCGTG2AALCQIFDSIFM1DVPMSRVKFNVNTEYA
          	***** ..     ..*****:**:****** *****::***:  *****:******:** 

MG05427.1 	YIQNFKVLQN1TFTKHQVDKPIPVAGLVKCKMQDNLEFLQWVKRFWDQYYPGGEYDAVAR
NCU00243.1	YIQNFKILQN1TFTKHQIEKSIPIEALVKCKMQDNLDFLQWTKRFWDQYYPGGDYDAAAR
FG06627.1 	YIQNFKVLQN1TFAKHQIDKPIPVPSLIKCKMQDNLEFLQWTKRFWDLNFPDHEYDAVSR
AN2862.1  	YLQNFKILQN1VFARHQIDKPIPVESLSKCRMQDNLEFLQWTKKYWDQHYPGGDYDALAR
          	*:****:*** .*::**::*.**: .* **:*****:****.*::**  :*. :*** :*

MG05427.1 	RKGAPLGAAGGGGGGAAVRAPVAGGAARRAGGTTPTTGAARAGL----GVSKANPALAQE
NCU00243.1	RKGGALPAT----MSGASRASAGSAAARRPGGATPT-GGPRVAARAPAANSAATQALQQE
FG06627.1 	RKGGSMPPS----AAAPKPVSATTGAARR-GGSTPS-GGPRVAK----AAGPATAALQQE
AN2862.1  	RKASGAPPG----SAGTSRAGATSASSAR-RGTTPTVGRARPAAGG--ASSATVAALQAE
          	**..   .      ...  . .  .:: *  *:**: * .* .  .  . . :  **  *

MG05427.1 	NATLKETVVGLERERDFYFSKLRDIELLVQNAVEEDPEIEKQEDGLVKQIQAILYSTE~E
NCU00243.1	VATLKEAVGGLERERDFYFHKLRDIEVLVQSAVEEDPELEKQEDGLIKAIQAILYSTE~E
FG06627.1 	NATLKETVVGLERERDFYFSKLRDIELLVQTAVDEDPELEKLEDGLVKQIQTILYSTE~E
AN2862.1  	LNSTKEAISGLEKERDFYFAKLRDIELLLQNAIEADPELEKDDDSLVKHIQGILYSTE0E
          	  : **:: ***:****** ******:*:*.*:: ***:** :*.*:* ** ****** *

MG05427.1 	GFEIPAEG--EVDDQETF
NCU00243.1	GFEIPEADAEAADDQETF
FG06627.1 	GFEIPAEG--EVDDQETF
AN2862.1  	GFEIPAEGEVAGDELETF
          	*****  .    *: ***
```
